# Having children with different men and subsequent cancer risk. A nationwide study in Denmark

**DOI:** 10.1038/sj.bjc.6601666

**Published:** 2004-02-24

**Authors:** R Campi, M Frydenberg, O Basso, P Ebbesen, J Olsen

**Affiliations:** 1 The Danish Epidemiology Science Centre, University of Aarhus, Vennelyst Boulevard 6, 8000 Aarhus C, Denmark; 2 Laboratory for Mother and Child Health, Istituto Ricerche Farmacologiche ‘Mario Negri’, Via Eritrea 62, 20157 Milan, Italy; 3 Department of Biostatistics, University of Aarhus, Vennelyst Boulevard 6, 8000 Aarhus C, Denmark; 4 Laboratory for Stem Cell Research, Aalborg University, Gustav Wieds Vej 10, 8000 Aarhus C, Denmark

**Keywords:** multiple partners, cancer, foetal antigens, pregnancy

## Abstract

The more men by whom a woman has children, the more diverse will be the foetal antigens of paternal origin introduced into her bloodstream, and we investigated whether this has an impact on subsequent cancer risks. By using population registries we identified 64 704 women who had children with at least two different partners from 1973 to 1996 in Denmark. We compared their cancer incidence with that of women who during the same time period had at least two births with no indication of partner change, adjusting for age, parity, socioeconomic factors and residence. The overall cancer incidence was more than 50% higher in women with two or more partners. Women having children with multiple partners had a higher incidence of cancer of the cervix and corpus uteri, a lower incidence of melanoma but a similar incidence of breast and ovarian cancer. Uncontrolled differences in lifestyle factors may explain the higher cancer risk associated with having multiple partners. The strong protective effect for melanoma was unexpected and deserves further study.

A protective effect of pregnancy on cancer is best documented for breast cancer (Rossing *et al*, 1996; Lambe *et al*, 2001) and ovarian cancer (Mockett *et al*, 2000). Other studies suggest a similar effect on colorectal (Talamini *et al*, 1998) and uterus cancers (Hinkula *et al*, 2002).

Associations with parity may be caused by pregnancy-induced hormonal changes, but could also have an immunological basis if women are immunised by the antigens of their fetuses (Janerich, 1980). Certain studies have provided evidence of immunization to antigens found in breast, ovarian and endometrial cancer cells (Janerich, 2001, 1994). Sera from multiparous women have been shown to react against antigens of ovarian cancer, in contrast to that from healthy nulliparous women. Foetal antigens, perhaps of parental origin, may protect against cancer (Shields *et al*, 1997). If so, such cancers should show some correlation with the number of pregnancies as well as with the number of different fathers of these pregnancies. Each new pregnancy results in an expression of new paternal genes, but a change of partner increases the genetic variation.

A reproductive history may, on the other hand, correlate with disease due to confounding. A level of health is required to carry a pregnancy to term (the ‘healthy pregnancy effect’), although we expect this to have only a limited effect on cancer risk.

We examined if having pregnancies with multiple partners influences the risk of some of the commonest cancers.

## MATERIAL AND METHODS

We conducted a historical follow-up study using data from the following population based registries: the Fertility Database, the Prevention Registry (each a combination of several registers), the National Hospital Discharge Registry, the Cause of Death Registry and the Cancer Registry. All the data for this study are located at Statistics Denmark or at the National Board of Health.

By means of the above-mentioned registers we identified a cohort comprising all (64 704) women with Danish citizenship who had had at least two births by at least two different men, and whose second birth took place between 1973 and 1996: the exposed cohort. We then selected a random sample of 100 000 women from the population of women with at least two children and who had their index birth (second birth or later) after 1973, as in the exposed cohort. Among these we selected the 86 624 women for whom there was no indication of a change of partner: the unexposed cohort. More details on the study population are provided elsewhere (Olsen *et al*, 2003).

These two cohorts were then linked to the cancer register and to the Population Register to obtain information on cancer incidence, death, emigration, women's education, social status, residence and occupation.

Incident diagnoses of cancer were extracted from the Cancer Register where they are coded according to the *International Classification of Diseases (ICD-7th Revision)*. Linkage was based upon the woman's civil personal registration number (CPR), since 1968 given to all residents in Denmark, and used in all public registers.

We analysed data using Poisson regression models. Exposure started at the first birth with a father different from the one registered at the previous birth(s). We considered women ‘exposed’ if they had at least one child by a different partner and if at least two different identifiable fathers were registered. Women who had a missing identifier for the father (2%) and no indication of a different father in any of the pregnancies were considered unexposed. Follow-up ended when cancer, death, or emigration occurred, or at the end of 1997 (when follow-up ended), whichever came first.

Missing data on social factors were divided into two categories. One consisted of unreported actual missing values (a), while the other consisted of values from years that lacked a classification, or changes to the classification over time (b). The cohorts were stratified by parity for which incidence rates over time are estimated and adjusted for potential confounders: parity (2, 3, 4 or more); number of partners (1, 2, 3 or more); current maternal age four age groups <25, 25–34, 35–44, >45 years); calendar period (1973–1980, 1981–1985, 1986–1990, ⩾1991); time since latest pregnancy (up to 10 years, more than 10 years); place of residence (Copenhagen; Aarhus, Odense; Aalborg; all other places; missing [a]; missing [b]); social status (self-employed, assisting spouses; high and medium workers; skilled workers; unskilled workers; unemployed, outside work; missing [a], missing [b]); education (basic school; high school; missing [a]; missing [b]); and 9) occupation (production industry; primary services; public and personal services; missing [a]; missing [b]).

If data were missing for the socioeconomic variables (people out of the workforce had no classification in some time periods), we used information from a previous year down to 1979. In the present analyses, missing data were kept in the analyses as a separate category, but all analyses were also performed after excluding subjects with missing data on social factors in the period 1973–1979. Analyses were performed using Poisson regressions in SAS and STATA software.

## RESULTS

Table 1

**Table 1:** Distribution of person years (PYs) according to potential confounders in the two cohorts

	**One partner**			**Two+ partners**		
**Variables**	**Total PYs**	**No. of cancer cases**	**IR per1000 PYs**	**Total PYs**	**No. of cancer cases**	**IR per 1000 PYs**
*Age during follow-up*						
<25	34048.0	127	3.73	24210.3	176	7.27
25–34	468241.1	1893	4.04	267043.9	2259	8.46
35–44	407856.8	1798	4.41	241769.9	1557	6.44
>45	35837.9	575	6.70	30031.1	181	6.02
						
*Place of residence*						
Copenhagen	52699.2	242	4.59	52708.3	346	6.56
Aarhus, Odense, Aalborg	85319.9	513	5.38	60265.1	559	9.28
All other places	755255.9	3322	4.40	436016.6	3203	7.35
Missing (a)	23563.2	87	3.69	4933.0	19	2.46
Missing (b)	69145.6	229	3.31	9192.1	46	3.75
						
*Education*						
Basic school	440434.2	2069	4.70	384028.2	2964	7.72
High school	160407.7	659	4.11	69227.4	482	6.96
Missing (a)	1058.5	2	1.89	1376.6	9	6.54
Missing (b)	394083.5	1663	4.22	108482.9	718	6.62
						
*Socioeconomic status*						
Self-employed assisting spouses	28454.4	137	4.65	13952.6	100	7.17
High/medium ranked workers	498394.9	2176	4.37	216828.7	1610	7.10
Skilled workers	21969.1	121	5.51	16292.5	151	9.27
Unskilled workers	189255.6	894	4.72	163084.6	1269	7.78
Outside work, unemployed	76164.9	372	4.88	110456.4	869	7.87
Missing (a)	53301.5	211	3.96	14330.3	82	5.72
Missing (b)	127443.5	482	3.78	18170.1	92	5.06
						
*Occupation*						
Production industry	132429.8	607	4.58	94382.1	718	7.61
Primary services	188050.4	826	4.39	113426.7	884	7.79
Public/personal services	459256.6	2128	4.63	291978.8	2151	7.37
Missing: (a)	88843.0	350	3.94	45184.0	328	7.26
Missing (b)	127403.9	482	3.78	18143.6	92	5.07
						
Total	995984.0	4393	4.41	563115.0	4173	7.41

^a^Unreported

^b^Lack of classification or modified classification.

shows details of the person years of observation and cancer observed. Altogether, 4173 events of cancer occurred in the cohort of women with two or more partners, while 4393 events were recorded among unexposed women.

As expected from their social profile, women with multiple partners had a higher risk of cancer of the cervix, but they also showed a higher incidence of cancer of the corpus uteri (Table 2

**Table 2:** Relative risk of specific cancers and cancers in general for women who have had children with different fathers

	**One partner**	**Two+ partners**				
Cancer site	**Cases**	**Cases**	**RR^a^**	**CI**	**RR^b^**	**95% CI**
Breast^c^	624	256	0.69	0.80	0.81^d^	0.77–1.07
Ovarian^e^	101	45	0.75	1.08	0.88	0.60–1.28
Cervix^f^	2783	3470	2.21	2.32	2.04	1.93–2.15
Uterus and corpus uteri^g^^,^^h^	43	38	1.49	2.37	1.84	1.14–2.96
Melanoma^i^	179	66	0.62	0.83	0.65	0.48–0.88
Lung^j^	50	34	1.15	1.81	1.45	0.89–2.36
Colon and rectum^k^	74	28	0.64	1.00	0.85	0.52–1.36
The rest of neoplasms^l^	663	342	0.87	1.00	1.00	0.87–1.15
						
All cancers	4393	4173	1.68	1.75	1.66	1.59–1.74

a Unadjusted.

b Adjusted for age, parity, time since latest pregnancy, place of residence, education, socioeconomic status, occupation.

c ICD7: 170, 470, 570, 670, 770, 870, 970.

d As in footnote b, plus adjustment for age at first delivery.

e ICD7: 175, 475, 575, 975.

f ICD7: 171, 471, 571, 771, 971.

g ICD7: 173, 473, 573, 873, 973.

h ICD7: 172, 472, 572, 872, 972.

i ICD7: 190, 590, 690, 990.

j ICD7: 162, 462.

k ICD7: 153, 253, 453, 553.

l ICD7: the rest.

). The relative risks of breast and ovarian cancer were less than one, but confidence limits included unity. For breast cancer the crude estimates were significantly less than one, but adjustment for age, parity and social factors reduced the effect and a statistically significant protective effect of having children by more than one partner was only seen for melanoma.

The parity specific partner effects are displayed in greater detail in Table 3

**Table 3:** Adjusted incidence rate ratios^a^ for the main cancer groups according to parity and number of partners. The reference category is that of women of parity 2 with one partner

	**Parity 2**		**Parity 3**				**Parity 4+**			
	**Partners**		**Partners**				**Partners**			
	**2**		**2**		**3**		**2**		**3+**	
**Cancer site**	**RR**	**CI 95%**	**RR**	**CI 95%**	**RR**	**CI 95%**	**RR**	**CI 95%**	**RR**	**CI 95%**
Breast^b^	0.99	0.79–1.24	0.84	0.63–1.12	1.01	0.60–1.69	0.49	0.26–0.90	0.74	0.33–1.65
Ovary	1.23	0.77–1.96	0.57	0.27–1.21	0.53	0.12–2.29	0.43	0.11–1.70	0.40	0.05–3.39
Cervix	2.00	1.87–2.13	2.23	2.00–2.49	2.75	2.36–3.20	0.97	0.76–1.25	2.06	1.60–2.65
Uterus and corpus uteri	2.38	1.32–4.29	1.70	0.67–4.30	0.96	0.12–7.80	0.53	0.09–3.20	0.58	0.06–6.02
Melanoma	0.70	0.47–1.03	0.68	0.40–1.15	0.51	0.15–1.66	0.22	0.05–1.06	0.56	0.11–2.83
Lung	1.63	0.87–3.06	1.72	0.69–4.27	2.54	0.78–8.29	NA^c^	—	0.82	0.14–4.72
Colon and rectum	0.85	0.43–1.66	0.92	0.42–2.01	0.72	0.22–4.24	0.35	0.23–2.06	1.21	0.21–6.91

a Adjusted for age, time since latest pregnancy, place of residence, education, socioeconomic status and occupation.

b Adjusted as in footnote ^a^ plus age at first delivery.

c There were no cases among women with two partners.

: no ‘dose–response’ effect with number of partners is shown for any of the cancers under study, including melanoma.

Malignant melanoma showed a nonsignificant association with parity, the adjusted rate ratio for parity being 1.04 (0.73–1.49), compared women with parity 2, and the rate ratio for parity 4 or higher was 0.65 (0.31, 1.37).

The effect of time interval since having a child by a different partner is shown in Table 4

**Table 4:** Relative incidence rate ratio for the main cancer grounds^a^ by time since last pregnancy for women who had children with two or more partners

	**Time since latest pregnancy**			
	**0–9 years**		**10+ years**	
**Cancer site**	**RR**	**95% CI**	**RR**	**95% CI**
Breast	0.92	0.72–1.15	0.87	0.68–1.11
Ovary	0.76	0.45–1.23	1.12	0.63–1.98
Cervix	2.08	1.96–2.21	1.79	1.57–2.04
Uterus and corpus uteri	1.85	1.05–3.27	1.66	0.69–4.00
Melanoma	0.63	0.44–0.89	0.75	0.42–1.34
Lung	1.41	0.68–2.91	1.44	0.75–2.77
Colon and rectum	0.89	0.46–1.71	0.81	0.40–1.63

a Adjusted for parity, age at first delivery, age, time since latest pregnancy, place of residence, education, socioeconomic status, occupation, reference group, women with two or more children with the same partner.

: no significant change in relative incidence rates was found for any cancer.

As breast cancers with an early onset may have a different aetiology than those with a later onset, we estimated the relative risks separately for women prior to 45 years of age and for women aged 46 years or older. When adjusted only for parity, both estimates suggested a protection of partner change (RR=0.80 (95% CI: 0.67–0.95) for women below 46 years and RR=0.68 (95% CI: 0.49–0.95) for older women). When age, age at first delivery, place of residence, education, socioeconomic status and occupation were included in the model, however, there was no significant reduction among younger women (RR=0.94 (95% CI: 0.78–1.14)) and only a nonsignificant decrease remained among older women (RR=0.72 (95% CI: 0.50–1.03)) (raw data not shown).

## DISCUSSION

This study did not provide strong support for the hypothesis that having children by different partners reduces the risk of breast or ovarian cancer (Janerich, 1980, 1994; Shields *et al*, 1997; Talamini *et al*, 1998; Lambe *et al*, 2001). We did see a statistically significant lower relative risk for breast cancer in the crude estimate, but the significance disappeared after adjustment. We have previously shown (Olsen *et al*, 2003) that adjustment for confounders is important. Previous findings on a reduced risk of breast cancer among women having children by different partners may thus have been subject to residual confounding (Janerich, 1980, 1994; Lambe *et al*, 2001).

We do not believe that the foetal antigen hypothesis explains the higher risk of cancer of the cervix. Rather the excess risk probably reflects confounding by lifestyle factors such as sexual habits.

The lower risk of melanoma related to two or more partners is novel and of interest because residual confounders would probably bias the effect measures towards higher values for this cancer. The result was unexpected, but rather strong. Melanoma is a common cancer in populations with fair skin and frequent exposure to sunlight. Two high-risk susceptibility genes have been identified (CDKN2A and CDK4) and gene–environment interactions may occur at several steps in cancer development (MacKie, 2002). Foetal antigens could in principle modify these gene–environmental interactions in different ways and if such a mechanism is important, the observed correlation with parity would be predicted.

The study has strengths as well as weaknesses. The strongest features lie in its population-based design with complete follow-up, reliable data on cancer incidence and reproduction, and independently reported paternal identity for live born children. We also had data on sociodemographic conditions of potential importance. All data were recorded independently of the study hypothesis, which might otherwise produce differential misclassification.

Without access to the participants we have, on the other hand, no possibility of verifying data on paternity and we have no data on pregnancies that ended with foetal loss. Our data refers to the putative biological father in official statistics but anecdotal evidence suggests that this may be in error in 1–10% of the cases. Misclassification of fathers may bias the effect measure for malignant melanoma, though we consider the magnitude of bias to be small.

If the foetal antigen hypothesis is true, the effect on parity level and number of partners is likely to be small. Although we included all women with two or more births by different partners into the study, our power to detect a small effect was limited as indicated by the wide confidence limits. It would be of interest to have data with longer follow-up time.

We found a protective effect of parity of having children with different men for malignant melanoma. Whether transmission of foetal cells to the mother's blood stream during pregnancy plays a role for this finding needs further studies.
